# Deficiency of Nuclear Receptor Coactivator 3 Aggravates Diabetic Kidney Disease by Impairing Podocyte Autophagy

**DOI:** 10.1002/advs.202308378

**Published:** 2024-03-14

**Authors:** Yaru Xie, Qian Yuan, Xinyi Cao, Yang Qiu, Jieyu Zeng, Yiling Cao, Yajuan Xie, Xianfang Meng, Kun Huang, Fan Yi, Chun Zhang

**Affiliations:** ^1^ Department of Nephrology, Union Hospital, Tongji Medical College Huazhong University of Science and Technology Wuhan 430000 China; ^2^ Department of Neurobiology, Institute of Brain Research, School of Basic Medical Sciences, Tongji Medical College Huazhong University of Science and Technology Wuhan 430000 China; ^3^ Tongji School of Pharmacy, Tongji Medical College Huazhong University of Science and Technology Wuhan 430000 China; ^4^ The Key Laboratory of Infection and Immunity of Shandong Province Department of Pharmacology School of Basic Medical Sciences Shandong University Jinan 250100 China

**Keywords:** autophagy, diabetic kidney disease, nuclear receptor coactivator, peroxisome proliferator‐activated receptor ‐γ, podocyte

## Abstract

Nuclear receptors (NRs) are important transcriptional factors that mediate autophagy, preventing podocyte injury and the progression of diabetic kidney disease (DKD). However, the role of nuclear receptor coactivators that are powerful enhancers for the transcriptional activity of NRs in DKD remains unclear. In this study, a significant decrease in Nuclear Receptor Coactivator 3 (NCOA3) is observed in injured podocytes caused by high glucose treatment. Additionally, NCOA3 overexpression counteracts podocyte damage by improving autophagy. Further, Src family member, Fyn is identified to be the target of NCOA3 that mediates the podocyte autophagy process. Mechanistically, NCOA3 regulates the transcription of Fyn in a nuclear receptor, PPAR‐γ dependent way. Podocyte‐specific *NCOA3* knockout aggravates albuminuria, glomerular sclerosis, podocyte injury, and autophagy in DKD mice. However, the Fyn inhibitor, AZD0530, rescues podocyte injury of *NCOA3* knockout DKD mice. Renal NCOA3 overexpression with lentivirus can ameliorate podocyte damage and improve podocyte autophagy in DKD mice. Taken together, the findings highlight a novel target, NCOA3, that protects podocytes from high glucose injury by maintaining autophagy.

## Graphical abstract

The study delves into the intricate mechanisms through which Nuclear receptors (NRs) co‐activator, Nuclear Receptor Coactivator 3 (NCOA3), contributes to the regulation of podocyte functions, emphasizing their crucial role in orchestrating the delicate balance essential for podocyte autophagy. The findings not only advance the understanding of the molecular intricacies underlying podocyte health but also provide potential therapeutic targets for the pathogenesis of diabetic kidney disease (DKD).

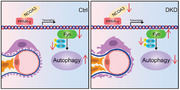

## 1 Introduction

Diabetic kidney disease (DKD) is characterized by impaired renal function and/or elevated urinary albumin excretion.^[^ ^1^ ^]^ Approximately 40% of patients with diabetes mellitus will develop DKD, even end‐stage renal disease.^[^ ^2^ ^]^ The key mechanism driving the progression of DKD is podocyte injury, which plays a pivotal role in mediating urinary albumin excretion, glomerulosclerosis, and the decline of renal function.^[^ ^3^ ^]^ Podocytes are terminally differentiated epithelial cells of the renal capsule, equipped with primary, secondary, and tertiary foot processes. They attach to the outer surface of the glomerular basement membrane (GBM) and, together with endothelial cells and the GBM, form the glomerular filtration barrier.^[^ ^4^ ^]^ In response to stimuli such as high glucose, podocytes experience injuries that manifest as foot‐process flattening, widening, and retraction, ultimately disrupting the integrity of the glomerular filtration barrier.^[^ ^5^ ^]^ This disruption can lead to severe consequences. For instance, when more than 20% of podocytes are lost due to detachment or death, irreversible changes occur, such as glomerulosclerosis and tubular interstitial fibrosis, further exacerbating DKD pathogenesis.^[^ ^3^, ^6^ ^]^ In summary, podocyte injury serves as the driving force behind the progression of DKD.

Autophagy plays a crucial role in maintaining cellular homeostasis by balancing the synthesis, degradation, and recycling of cellular proteins and organelles, ultimately providing essential nutrients for cell survival.^[^ ^7^ ^]^ This process involves a cascade of autophagy‐related (Atg) proteins that form a complex responsible for catalyzing the conversion of LC3‐I to LC3‐II, which then inserts into the autophagosome. Subsequently, the autophagosome fuses with the lysosome, facilitating the degradation of superfluous proteins and organelles.^[^ ^8^ ^]^ Podocytes, as specialized cells in the kidney, exhibit a significantly high basal autophagic activity, underscoring the critical role of autophagy in maintaining their cellular homeostasis.^[^ ^9^ ^]^ However, in both DKD patients and mice, podocyte autophagy is substantially impaired.^[^ ^10^, ^11^ ^]^ This impairment has been associated with worsened podocyte injury, proteinuria, and glomerulosclerosis in DKD mice and aging mice when podocyte‐specific *Atg5* knockout is induced.^[^ ^10^, ^12^ ^]^ Conversely, increased podocyte autophagy has been shown to confer protection against podocyte injury induced by high glucose treatment.^[^ ^13^ ^]^ Gaining a deeper mechanistic understanding of the pathophysiology of podocyte autophagy holds great potential in addressing podocyte injury and advancing the development of novel therapies for treating DKD patients.

Nuclear receptors (NRs) are a family of transcriptional factors that regulate a broad spectrum of physiological and pathological functions including autophagy.^[^ ^14^ ^]^ NRs such as Vitamin D receptor (VDR), estrogen receptor, retinoic acid receptor‐α, Peroxisome proliferator‐activated receptor‐γ (PPAR‐γ), and mineralocorticoid receptor have been identified to be crucial in podocyte injury.^[^ ^15^ ^]^ As transcriptional factors, NRs always need coactivators to enhance transcriptional activity. However, the coactivators of NRs in kidney disease have received limited attention. The Nuclear receptor coactivator (NCOA) family which includes NCOA1, NCOA2, and NCOA3, serves as transcriptional coactivators for NRs and other transcription factors.^[^ ^16^ ^]^ The presence of multiple LXXLL motifs on NCOAs facilitates their physical binding with agonist‐bound NRs, forming NRs boxes.^[^ ^17^ ^]^ NCOAs have been implicated in various diseases, such as cancer, arthritis, and immune response. However, its role in kidney diseases remains unclear.

In this study, we found that NCOA3 but not NCOA1 and NCOA2 decreased obviously in diabetic kidneys. Overexpression of NCOA3 with lentivirus inhibited podocyte injury and improved podocyte autophagy in vitro. Mechanistically, NCOA3 downregulated the transcription of a member of the src kinase family, Fyn in a PPAR‐γ dependent way. Fyn knockdown counteracted the decreased autophagy induced by NCOA3 deficiency. To verify the effect and mechanism of NCOA3 in vivo, we generated podocyte‐specific knockout mice. NCOA3 knockout aggravated podocyte injury, urinary albumin excretion, and glomerulosclerosis, and led to decreased autophagy in the DKD model. Fyn inhibitor, AZD0530 attenuated glomerular and podocyte injury of podocyte‐specific *NCOA3* knockout DKD mice. Further, we demonstrated that NCOA3 overexpression ameliorated the progression of DKD in vivo. In conclusion, our study sheds new light on the significance of NCOA3 in regulating podocyte autophagy, offering a promising therapeutic target for treating DKD.

## 2 Results

### 2.1 NCOA3 was Reduced in the Kidneys of Diabetic Mice and Patients

**Figure 1:**
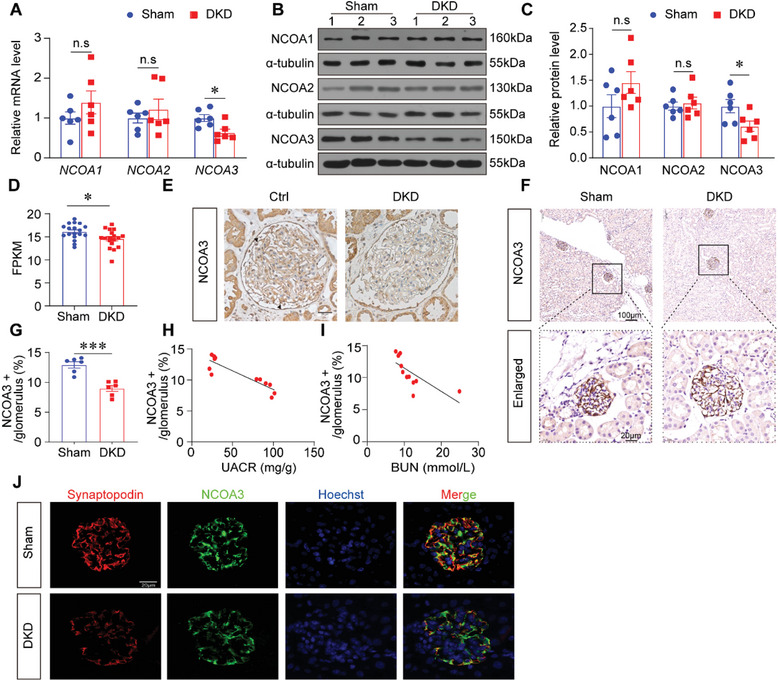
NCOA3 was dramatically downregulated in the kidneys of DKD mice. A) Relative mRNA levels of *NCOA1*, *NCOA2*, and *NCOA3* in Sham and DKD mice (*n* = 6 per group). B,C) Representative Western blotting (B) and quantification (C) of NCOA1, NCOA2, and NCOA3 show relative protein levels in the kidney in different groups (*n* = 6 per group). D) Expression levels from GEO datasets (GSE217853) showed NCOA3 expression in Sham mice and DKD mice. E) Representative immunohistochemical staining of NCOA3 in the kidneys of control individuals and patients with DKD. Scale bar, 20 µm. F,G) Representative immunohistochemical staining images of NCOA3 (F) and quantified date (G) showing the levels of NCOA3 in glomerular from Sham and DKD mice (15 glomeruli per mouse, *n* = 6 per group). Scale bar, 100 µm (top), 20 µm (bottom). H) Negative correlation between the NCOA3‐positive stained area quantification and UACR in all mice (Pearson r = −0.8845, *P* = 0.0002, *n* = 12 mice). I) Negative correlation between quantification of the NCOA3‐positive stained area and BUN in all mice (Pearson r = −0.7, *P* = 0.0113, *n* = 12 mice). J) Representative immunofluorescence images of NCOA3 (green) and Synaptopodin (red) in the podocytes from Sham and DKD mice. Scale bar, 20 µm. * *P* < 0.05, *** *P* <0.001, n.s, no significance. Data are expressed as mean ± standard error of the mean (SEM). The data distribution was normal, and Student's t‐test was applied for comparisons between groups.

We first screened the levels of nuclear receptor coactivator (NCOA) family members in the kidneys of Sham and diabetic kidney disease (DKD) mice. The mRNA level of *NCOA3* was significantly decreased in DKD group, whereas *NCOA1* and *NCOA2* exhibited comparable levels to the Sham group (**Figure** **1**A). Consistent results were obtained from Western blotting analyses (Figure 1B,C). Then we performed bioinformatics analysis of public DKD datasets (GSE217853) and identified the lower expression of NCOA3 in the kidneys of DKD mice (Figure 1D). Based on these findings, we proceeded to investigate the expression pattern of NCOA3 in human kidney biopsies. Immunohistochemical staining revealed that NCOA3 was abundantly expressed in healthy glomeruli, while its level was significantly reduced in DKD patients (Figure 1E). The immunohistochemical staining in DKD mice showed consistent results (Figure 1F,G). Importantly, NCOA3 expression was negatively correlated to the urine albumin to creatinine ratio (UACR) (Figure 1H) and blood urea nitrogen (BUN) level (Figure 1I). We further demonstrated the location of NCOA3 on podocytes by co‐staining NCOA3 with the marker of the podocyte, Synaptopodin. These results indicated that NCOA3 was more abundant in podocytes from control mice compared to DKD mice (Figure 1J). Additionally, when the human podocyte cells (HPCs) were treated with high glucose (HG) for 12 hours or 24 hours, the protein level of NCOA3 was significantly downregulated (Figure S1A,B, Supporting Information). In order to get rid of the effect of osmotic pressure on podocytes, mannitol was added in the medium as the osmotic pressure control for HG, there was no difference between the control group and the mannitol group (Figure S1C,D, Supporting Information). Then, we detected the NCOA3 levels in HPCs with advanced glycation end products (AGE), our results revealed that AGE stimuli significantly reduced NCOA3 expression in a concentration‐dependent manner (Figure S1E,F, Supporting Information). These results indicated that the expression of NCOA3 was at a high level in healthy podocytes, however, decreased greatly when suffering from high glucose.

### 2.2 NCOA3 Protected Against Podocyte Injury by Maintaining Autophagy

**Figure 2:**
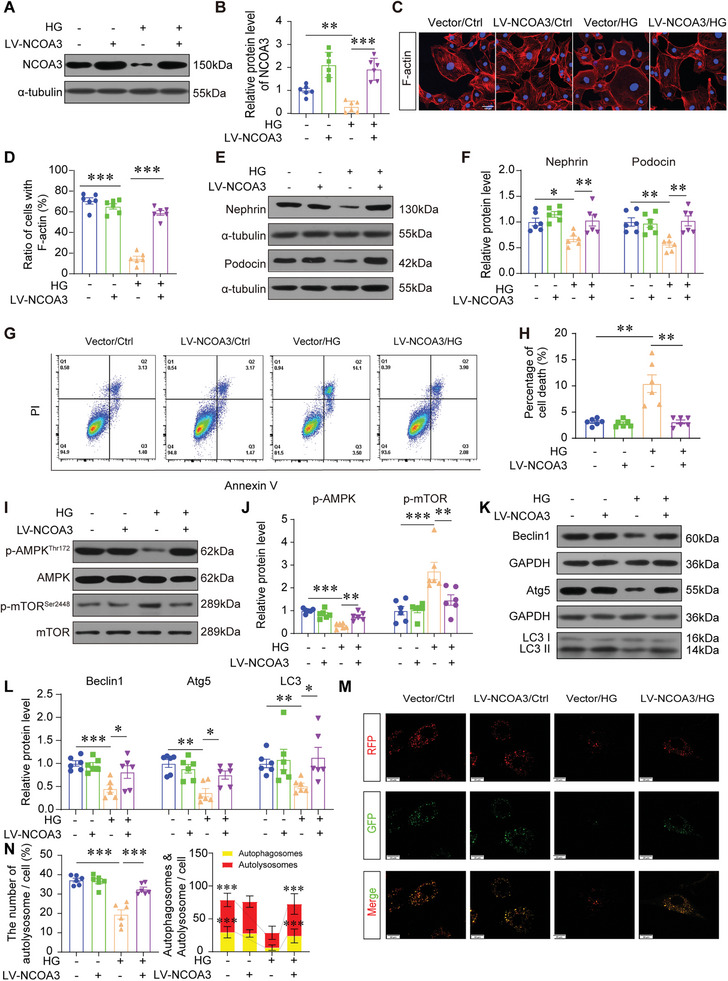
NCOA3 protected against podocyte injury by maintaining autophagy. A,B) Representative Western blotting (A) and quantification (B) of NCOA3 in human podocyte cell lines treated with NCOA3 lentivirus (LV‐NCOA3) transfection and high glucose (HG) stimulation (*n* = 6). C,D) Representative immunofluorescence images (C) and quantification (D) of F‐actin stained by phalloidin (*n* = 6). Scale bar, 100 µm. (E, F) Representative Western blotting E) and quantification F) of Nephrin and Podocin after HG stimulation for 24 h following LV‐NCOA3 transfection (*n* = 6). G,H) Level of apoptosis of podocytes under different treatment approaches detected by flow cytometry (*n* = 6). I,J) Representative Western blotting (I) and quantification (J) of phosphorylated AMP‐activated protein kinase (p‐AMPK), and phosphorylated mechanistic target of rapamycin (p‐mTOR) after HG stimulation for 24 h with LV‐NCOA3 transfection (*n* = 6). K,L) Representative Western blotting (K) and quantification (L) of Beclin 1, Atg5, and LC3. M,N) Representative images of LC3 fluorescence in podocytes infected with GFP‐RFP‐LC3 adenovirus for 24 hours (M) and quantification of the proportion of autophagosomes quantity (N, left), and quantification of the number of autophagosomes (yellow) dots and autolysosome (red) dots (N, right) (*n* = 6). Scale bar, 20 µm. * *P* < 0.05, ** *P* < 0.01, *** *P* <0.001. Data are expressed as mean ± SEM. One‐way ANOVA was applied for comparisons.

Due to the diminished expression of NCOA3 in injured podocytes, we embarked on investigating whether NCOA3 overexpression could alleviate podocyte injury. Successfully, NCOA3 was overexpressed in podocytes through NCOA3 lentivirus (LV‐NCOA3) transfection, as evidenced in **Figure** **2**A,B. Subsequently, we observed that NCOA3 overexpression effectively improved actin cytoskeleton derangement (Figure 2C,D), reversed the reduction of Nephrin and Podocin, which were crucial for maintaining slit diaphragm integrity (Figure 2E,F), and even mitigated podocyte apoptosis after HG treatment (Figure 2G,H). Considering the well‐established role of autophagy disorder in mediating podocyte injury, we delved into exploring the impact of NCOA3 on podocyte autophagy.^[^ ^18^ ^]^ The Western blotting results showed NCOA3 overexpression effectively counteracted the decline in AMP‐activated protein Kinase (AMPK) activation and the elevation in mammalian target of Rapamycin (mTOR) activation induced by HG stimulation (Figure 2I,J). These changes are crucial regulatory processes involved in autophagy. Furthermore, NCOA3 overexpression was observed to restore autophagy in podocytes, as demonstrated by the levels of autophagy‐related proteins, such as Atg5, Beclin1, and LC3, which exhibited an increase following NCOA3 lentivirus transfection (Figure 2K,L). To further confirm the autophagy flux, we constructed a tandem GFP‐RFP‐LC3 adenovirus. The results showed that autophagy flux was blocked by HG treatment. However, NCOA3 overexpression promoted autophagy flux as revealed by the increased presence of red punctate cells (Figure 2M,N). In conclusion, these results indicated that NCOA3 prevented podocyte injury by promoting autophagy.

### 2.3 NCOA3 Modulated Podocyte Autophagy by Downregulating the Transcription of Fyn in PPAR‐γ Dependent Way

**Figure 3:**
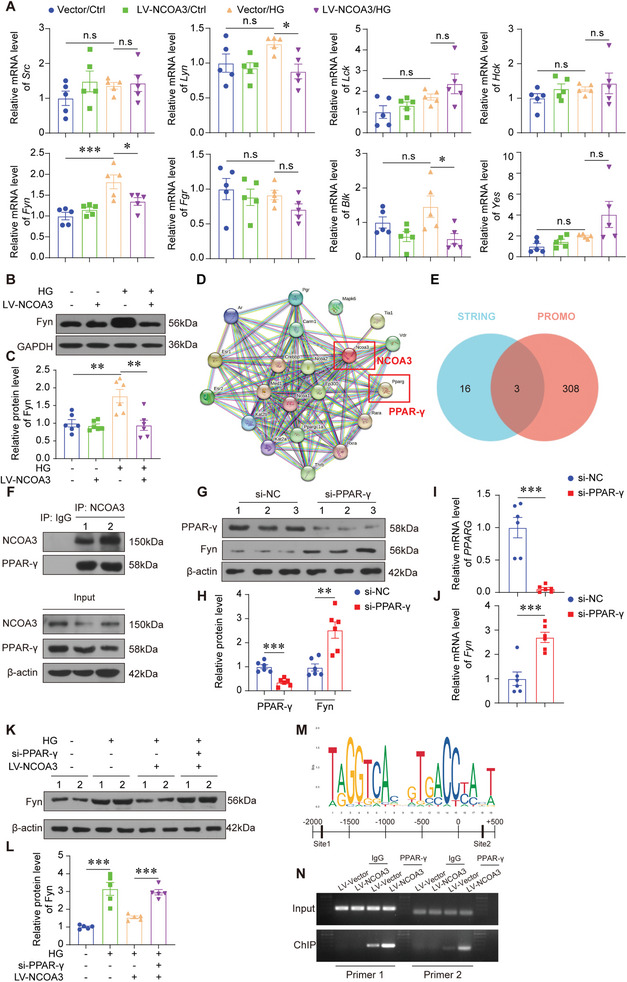
NCOA3 modulated podocyte autophagy by downregulating the transcription of Fyn in a PPAR‐γ dependent way. A) relative mRNA levels of SFKs (*Src*, *Lyn*, *Lck*, *Hck*, *Fyn*, *Fgr*, *Blk*, *Yes*) after HG stimulation for 24 h with LV‐NCOA3 transfection (*n* = 5 per group). B,C) Representative Western blotting (B) and quantification (C) of Fyn after HG stimulation for 24 h with LV‐NCOA3 transfection (*n* = 6). D) The potential target of NCOA3 predicted by STRING database. E) The Venn diagram of transcription factors of Fyn predicted by PROMO database (red) and interacted proteins of NCOA3 predicted by STRING database (blue). F) Co‐IP of NCOA3 and PPAR‐γ. G,H) Representative Western blotting (G) and quantification (H) of PPAR‐γ and Fyn with si‐ PPAR‐γ transfection (*n* = 6). I,J) Relative mRNA level of *PPARG* (I) and *Fyn* (J) with si‐PPAR‐γ transfection (*n* = 6). K,L) Representative Western blotting (K) and quantification (L) showing relative protein level of Fyn after HG stimulation for 24 h with LV‐NCOA3 and/or si‐ PPAR‐γ transfection (*n* = 5). M) The predictive motif of PPAR‐γ DNA‐binding domain from jaspar.genereg.net and the latent binding site of PPAR‐γ at the promoter of Fyn. Primer 1 was designed targeting to −1850 to −1833 (site 1), primer 2 was designed targeting to +372 to +391. N) Representative images of ChIP‐PCR in HPCs treated with LV‐NCOA3 or LV‐Vector transfection. * *P* < 0.05, ** *P* < 0.01, *** *P* <0.001, n.s, no significance. Data are expressed as mean ± SEM. One‐way ANOVA was applied for comparisons.

Src family kinases (SFKs) play a key role in regulating the autophagy of renal resident cells.^[^ ^19^ ^]^ Thus, we assessed the expression patterns of SFKs in the podocytes treated with HG with or without NCOA3 lentivirus. Our results showed that among SFKs, only the mRNA level of *Fyn* was induced by HG, and downregulated by NCOA3 overexpression significantly (**Figure** **3**A). Further, we confirmed that the elevated protein level of Fyn induced by HG was reduced by NCOA3 overexpression in HPCs (Figure 3B,C). As NCOA3 can't bind to the promoter of target genes and generally acts as a coactivator of nuclear receptors, we tried to find potential proteins interacted with NCOA3 by the STRING database (Figure 3D). And the transcription factors of Fyn were predicted with the PROMO database. Three nuclear receptors, Peroxisome proliferator‐activated receptor‐γ (PPAR‐γ), Vitamin D receptor (VDR), and androgen receptor (AR) that were predicted to be the transcriptional factor and interacted with NCOA3 were found (Figure 3E). PPAR‐γ was the most well‐studied nuclear receptor in podocyte biology.^[^ ^15^ ^]^ Thus, we further confirmed the binding between NCOA3 and PPAR‐γ using Co‐IP. PPAR‐γ was blotted in the complex immunoprecipitated with NCOA3 antibody (Figure 3F). And we detected the expression of Fyn regulated by PPAR‐γ. PPAR‐γ knockdown increased the mRNA level and protein level of Fyn (Figure 3G–J). At the same time, PPAR‐γ knockdown blocked the downregulation of Fyn by NCOA3 overexpression (Figure 3K,L). To explore whether PPAR‐γ regulates Fyn by binding its promoter, we found several DNA binding elements of PPAR‐γ on the Fyn promoter by analyzing the JASPAR database. Two pairs of primers were designed to identify these binding elements. Chromatin immunoprecipitation (ChIP) showed that PPAR‐γ bound to the promoter of Fyn, and NCOA3 overexpression promoted the interaction between them (Figure 3M,N). These results indicated that NCOA3 downregulated the transcription of Fyn in a PPAR‐γ dependent way.

**Figure 4:**
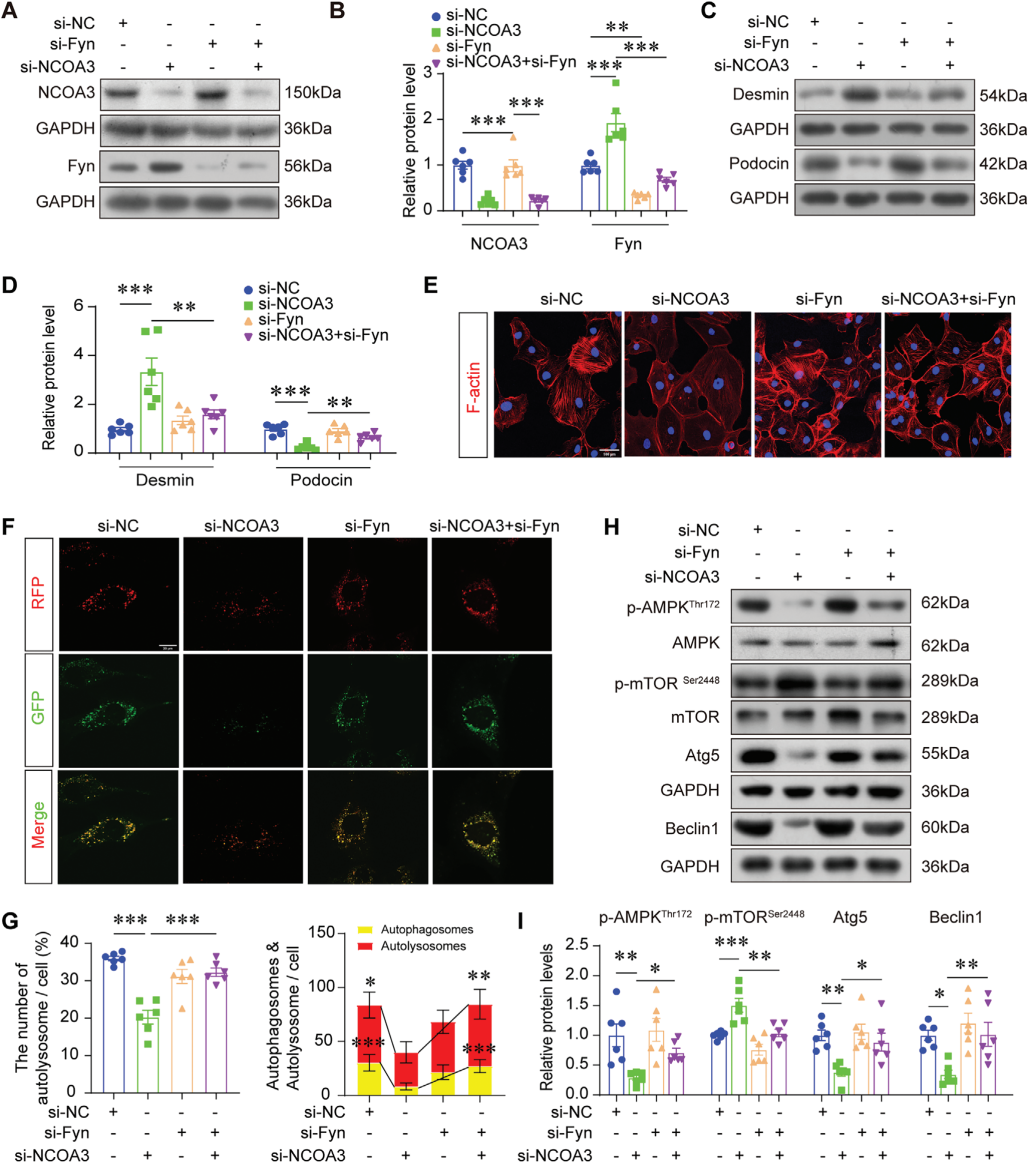
Inhibition of Fyn rescued the functional defect in *NCOA3*‐deficient podocytes. A,B) Representative Western blotting (A) and quantification (B) showing relative protein levels of NCOA3 and Fyn in podocytes treated with si‐NCOA3 and/or si‐Fyn transfection (*n* = 6). C,D) Representative Western blotting (C) and quantification (D) of Desmin and Podocin in podocytes under different treatments (*n* = 6). E) Representative microscopic images of F‐actin using phalloidin staining in podocytes. Scale bar, 100 µm. F,G) Representative images of LC3 fluorescence in podocytes infected with GFP‐RFP‐LC3 adenovirus for 24 hours (F) and quantification of the proportion of autophagosomes quantity (G, left), and quantification of the number of autophagosomes (yellow) dots and autolysosome (red) dots (G, right) (*n* = 6). Scale bar, 20 µm. H,I) Representative Western blotting (H) and quantification of p‐AMPK, p‐mTOR, Atg5, and Beclin1 (I) under different treatments (*n* = 6). * *P* < 0.05, ** *P* < 0.01, *** *P* <0.001. Data are expressed as mean ± SEM. One‐way ANOVA was applied for comparisons.

To demonstrate the indispensable role of Fyn in NCOA3 mediated autophagy, we inhibited Fyn expression in HPCs combined with NCOA3 knockdown. This silencing effect was validated by Western blotting (**Figure** **4**A,B). Fyn knockdown inhibited the upregulation of Desmin and the decrease of Podocin in NCOA3‐deficient podocytes (Figure 4C,D). Actin cytoskeleton derangement of podocytes caused by NCOA3 knockdown was ameliorated by Fyn deficiency (Figure 4E). Furthermore, Fyn silencing restored autophagic flux in NCOA3‐deficient podocytes (Figure 4F,G). The Western blotting revealed that the regulators of autophagy, phosphorylated AMPK was increased and phosphorylated mTOR was inhibited by Fyn knockdown. And the reduction of autophagy‐associated proteins like Atg5 and Beclin1 caused by NCOA3 knockdown was restored by Fyn knockdown (Figure 4H,I). All these results above‐mentioned suggested that NCOA3 promoted autophagy by downregulating Fyn.

To further confirm that NCOA3 promotes autophagy by inhibiting Fyn expression, we evaluated the effects of NCOA3 overexpression combined with Fyn knockdown on autophagy and podocyte injury under HG conditions and found that NCOA3 was overexpressed and Fyn was knockdown successfully (Figure S2A–C, Supporting Information), Fyn knockdown combined with NCOA3 overexpression further restored the upregulation of Desmin and reduction of Podocin in podocytes induced by HG stimuli, compared to overexpression of NCOA3 alone (Figure S2D–F, Supporting Information). And autophagy‐associated proteins like Atg5, Beclin1, p62, and LC3, which were restored by NCOA3 overexpression, were further recovered by Fyn knockdown (Figure S2G,H, Supporting Information). These results substantiate the assertion that NCOA3 promotes autophagy by suppressing Fyn expression.

### 2.4 Podocyte‐Specific *NCOA3* Deletion Aggravated Podocyte Injury of DKD Mice

To investigate the role of NCOA3 in podocytes in vivo, we hybridized NPHS‐Cre mice with *NCOA3^fl^/^fl^* mice to generate podocyte‐specific *NCOA3* knockout mice (Figure S3A,B, Supporting Information).^[^ ^20^ ^]^ Immunofluorescence staining (Figure S3C, Supporting Information) and Western blotting analysis of isolated glomeruli (Figure S3D–F, Supporting Information) showed NCOA3 on podocytes was knocked out successfully. We observed that *Cre^+^/NCOA3^fl^/^fl^* mice at 12 months old showed elevated albuminuria (Figure S3G, Supporting Information), podocyte deficiency (Figure S3H,I, Supporting Information), glomerulosclerosis (Figure S3J,K, Supporting Information), and podocyte damage as manifested by podocyte foot process broadening, glomerular basement membrane (GBM) thickening, and podocyte process disappearance, and loss of Nephrin (Figure S3I–O, Supporting Information). These results suggest that NCOA3 deficiency is a driving force of podocyte injury.

**Figure 5:**
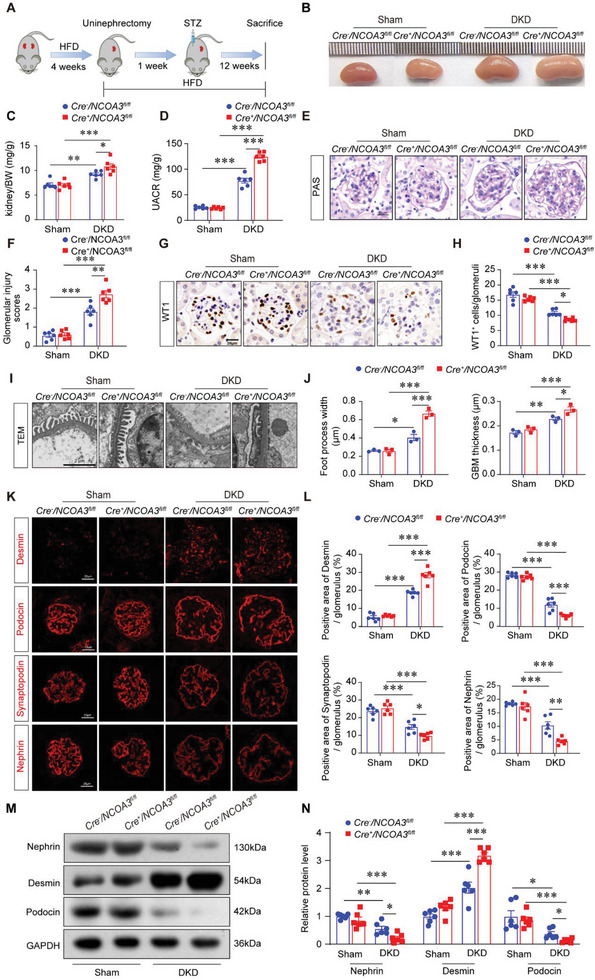
Podocyte‐specific *NCOA3* deletion exacerbated podocyte injury in DKD mice. A) Schematic showing the procedure of HFD/STZ induced DKD model. B) Representative kidney images of each group. C) The ratio of kidney weight to body weight (BW) (*n* = 6). D) UACR in mice in different groups (*n* = 6). E,F) Representative images of PAS staining (E) and glomerular injury scores (F) (15 glomeruli per mouse, n = 6 mice per group). Scale bar, 20 µm. G,H) Representative photomicrographs (G) and quantification (H) of WT1 showed the number of podocytes in each glomerular (15 glomeruli per mouse were analyzed, *n* = 6 mice per group). Scale bar, 20 µm. (I, J) Representative micrographs I) and quantification J) of foot process width and GBM thickness in different groups by TEM analyses (15 glomeruli per mouse, *n* = 3 mice per group). Scale bar, 2 µm. K,L) Representative immunofluorescence staining images (K) and quantification (L) of Desmin, Podocin, Synaptopodin, and Nephrin in the kidney from different groups (15 glomeruli per mouse, *n* = 6 mice per group). Scale bar, 20 µm. M,N) Representative Western blotting (M) and quantification (N) showing relative protein levels of Nephrin, Desmin, and Podocin in the glomeruli from different groups (*n* = 6 per group). * *P* < 0.05, ** *P* < 0.01, *** *P* <0.001. Data are expressed as mean ± SEM. One‐way ANOVA was applied for comparisons among the four groups.

To elucidate the role of NCOA3 in podocyte injury and DKD in vivo, the DKD model was established with *Cre^+^/NCOA3^fl/fl^* and *Cre^−^/NCOA3^fl/fl^* mice by high‐fat diet (HFD) followed by unilateral nephrectomy and streptozocin (STZ) injection, as illustrated in **Figure** **5**A. There were no significant differences in blood glucose and body weight between *Cre^+^/NCOA3^fl/fl^* and *Cre^−^/NCOA3^fl/fl^* DKD mice (Table S1, Supporting Information), however, *Cre^+^/NCOA3^fl/fl^* DKD mice exhibited enlarged kidneys (Figure 5B,C), increased UACR (Figure 5D) compared with *Cre^−^/NCOA3^fl/fl^* DKD mice. Periodic acid‐Schiff (PAS) staining was used to show glomerular mesangial expansion. As revealed by Figure 5E,F, glomerulosclerosis was more severe in the kidney of *Cre^+^/NCOA3^fl/fl^* DKD mice. Furthermore, podocyte injury was detected. Podocyte number reflected by WT1 immunohistochemical staining showed that podocyte‐specific *NCOA3* knockout promoted podocyte loss in DKD (Figure 5G,H). The foot process width of podocytes and GBM thickness were observed with the transmission electron microscope (TEM). The results showed foot process was broadened and GBM was thickened in *Cre^+^/NCOA3^fl/fl^* DKD mice compared with *Cre^−^/NCOA3^fl/fl^* DKD mice (Figure 5I,J). The protein level of podocyte injury marker, Desmin, slit diaphragm proteins like Podocin and Nephrin, and podocyte skeleton‐associated protein, Synaptopodin were further detected by immunofluorescence staining. NCOA3 knockout aggravated the increase of Desmin and the loss of Podocin, Synaptopodin, and Nephrin in DKD glomeruli (Figure 5K,L). Western blotting of Nephrin, Desmin, and Podocin verified these results (Figure 5M,N). These results indicated that the deletion of NCOA3 aggravated albuminuria, glomerulosclerosis, and podocyte injury.

### 2.5 Autophagy was Inhibited in the Glomeruli of Podocyte‐Specific *NCOA3* Knockout DKD Mice

**Figure 6:**
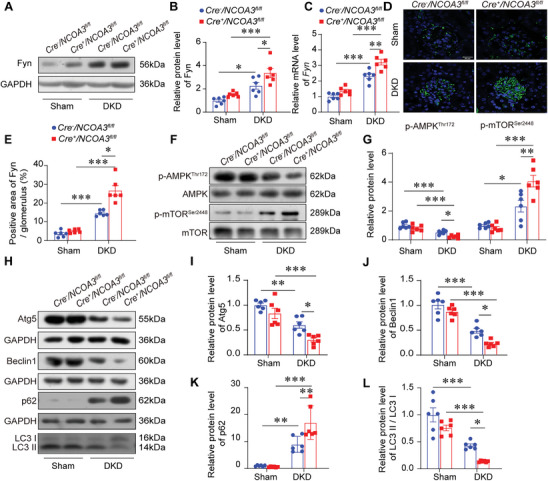
Autophagy was inhibited in the glomeruli of *Cre^+^/NCOA3^fl/f^* ^l^ DKD mice. A,B) Representative Western blotting (A) and quantification (B) of Fyn in the glomeruli from different groups (*n* = 6 per group). C) Relative mRNA level of *Fyn* in the kidneys from different groups (*n* = 6 per group). D,E) Representative immunofluorescence staining images (D) and quantification (E) showing the expression of Fyn in the glomeruli from different groups (15 glomeruli per mouse, *n* = 6 mice per group). Scale bar, 20 µm. F,G) Representative Western blotting (F) and quantification of p‐AMPK and p‐mTOR (G) in the glomeruli from different groups (*n* = 6). H–L) Representative Western blotting (H) and quantification showing relative protein levels of Atg5 (I), Beclin1 (J), p62 (K), and LC3 (L) in the glomeruli from different groups (*n* = 6 per group). * *P* < 0.05, ** *P* < 0.01, *** *P* <0.001. Data are expressed as mean ± SEM. One‐way ANOVA was applied for comparisons among the four groups.

To clarify the mechanism of NCOA3 in vivo, glomeruli were isolated to assess the expression of Fyn and autophagy. Compared to *Cre^−^/NCOA3^fl/fl^* DKD mice, the protein level (**Figure** **6**A,B) and mRNA level (Figure 6C) of Fyn was increased greatly in the glomeruli of *Cre^+^/NCOA3^fl/f^* ^l^ DKD mice, as well as the expression of Fyn in glomeruli as evidenced by immunofluorescence staining (Figure 6D,E). The positive regulator of autophagy, phosphorylated AMPK was downregulated, and the negative regulator of autophagy, phosphorylated mTOR was increased in the glomeruli of *Cre^−^/NCOA3^fl/f^* ^l^ DKD mice. However, these changes were promoted by podocyte‐specific *NCOA3* knockout (Figure 6F,G). Autophagy level reflected by the expression of Atg5, Beclin1, and LC3 II in *Cre^+^/NCOA3^fl/f^* ^l^ DKD mice was lower than *Cre^−^/NCOA3^fl/fl^* DKD mice, and the expression of p62 in *Cre^+^/NCOA3^fl/f^* ^l^ DKD mice was higher than *Cre^−^/NCOA3^fl/fl^* DKD mice (Figure 6H–L).

### 2.6 Fyn Inhibition Rescued NCOA3 Deficiency Induced Podocyte Injury in DKD Mice

**Figure 7:**
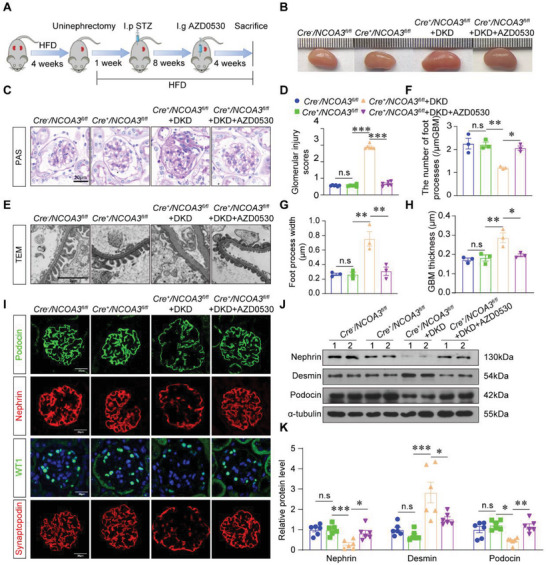
Fyn inhibition with AZD0530 rescued podocyte injury of *Cre^+^/NCOA3^fl/f^* ^l^ DKD mice. A) Schematic showing the procedure of the DKD model with AZD0530 injection. B) Representative overall kidney pictures of each group. C,D) Representative images (C) and glomerular injury scores (D) from PAS staining (15 glomeruli per mouse, *n* = 6 mice per group). Scale bar, and 20 µm. E–H) Representative micrographs (E) and quantification of the number of foot processes (F), foot process width (G), and GBM thickness (H) in different groups by TEM analyses (15 glomeruli per mouse, *n* = 3 mice per group). Scale bar, 2 µm. I) Representative immunofluorescent staining micrographs showing the expression of Podocin (green), Nephrin (red), WT1 (green), and Synaptopodin (red) in the kidney from different groups. Scale bar, 20 µm. J,K) Representative Western blotting (J) and quantification (K) of Nephrin, Desmin, and Podocin from different groups (*n* = 6 per group). * *P* < 0.05, ** *P* < 0.01, *** *P* <0.001, n.s, no significance. Data are expressed as mean ± SEM. One‐way ANOVA was applied for comparisons among the four groups.

To identify that Fyn mediates NCOA3 deficiency induced podocyte injury, we constructed a rescue animal model with AZD0530, an inhibitor of Fyn, in *Cre^+^/NCOA3^fl/f^* ^l^ DKD mice (**Figure** **7**A).^[^ ^21^ ^]^ The kidney enlargement was inhibited in *Cre^+^/NCOA3^fl/f^* ^l^ DKD mice treated with AZD0530 compared to *Cre^+^/NCOA3^fl/f^* ^l^ DKD mice (Figure 7B). As showed by PAS staining, glomerulosclerosis of *Cre^+^/NCOA3^fl/f^* ^l^ DKD mice was alleviated by AZD0530 treatment (Figure 7C,D). TEM results showed AZD0530 restored the number of foot processes, inhibited the expansion of the foot process, and decreased the GBM thickness of *Cre^+^/NCOA3^fl/f^* ^l^ DKD mice (Figure 7E–H). Podocyte damage revealed by Podocin, Nephrin, and Synaptopodin, and podocyte number reflected by WT1 in *Cre^+^/NCOA3^fl/f^* ^l^ DKD mice were recovered by AZD0530 (Figure 7I). The consistent results were shown by Western blotting that the expression of Nephrin and Podocin was increased and the expression of Desmin was inhibited by AZD0530 (Figure 7J,K). Thus, Fyn plays an essential role in mediating podocyte injury caused by NCOA3 knockout.

### 2.7 NCOA3 Overexpression Attenuated Glomerular and Podocyte Injury, and Improved Autophagy in the Kidneys of DKD Mice

**Figure 8:**
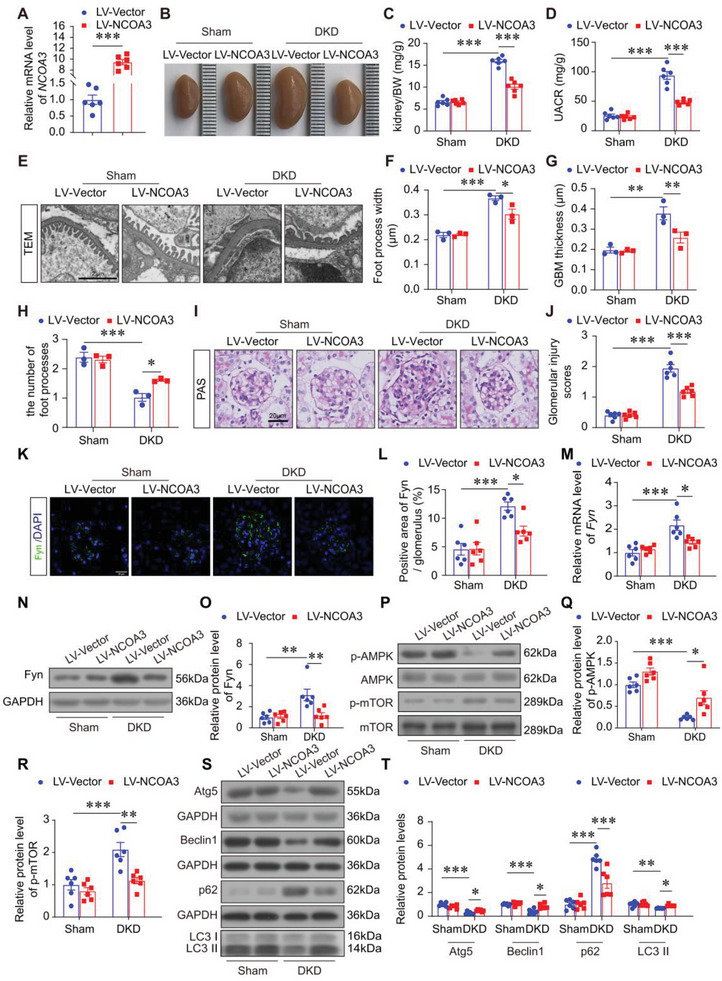
NCOA3 protected against DKD in vivo. A) The relative mRNA level of *NCOA3* in isolated glomeruli at 4 weeks after LV‐NCOA3 injection into mice kidneys. B) Representative overall kidney pictures of each group. C) The ratio of kidney weight to body weight (BW) (*n* = 6). D) The level of UACR in different groups (*n* = 6). E–H) Representative micrographs (E) and quantification of foot process width (F), GBM thickness (G), and the number of foot processes (H) in different groups by TEM analysis (15 glomeruli per mouse, *n* = 3 mice per group). Scale bar, 2 µm. I,J) Representative images (I) and glomerular injury scores (J) from PAS staining (15 glomeruli per mouse, n = 6 mice per group). Scale bar, and 20 µm. K,L) Representative immunofluorescence images (K) and quantification (L) of Fyn (green) in the glomeruli from different groups (15 glomeruli per mouse, n = 6 mice per group). Scale bar, 20 µm. M) Relative mRNA level of *Fyn* in the kidneys from different groups (*n* = 6 per group). N,O) Representative Western blotting (N) and quantification (O) of Fyn in the kidneys from different groups of mice (n = 6 per group). P–R) Representative Western blotting (P) and quantification showing relative protein levels of p‐AMPK (Q), and p‐mTOR (R) in the kidneys from different groups (*n* = 6 per group). S,T) Representative Western blotting (S) and quantification of Atg5, Beclin1, p62, and LC3 (T) in the kidneys from different groups (n = 6 per group). * *P* < 0.05, ** *P* < 0.01, *** *P* <0.001. Data are expressed as mean ± SEM. One‐way ANOVA was applied for comparisons among the four groups.

To determine whether NCOA3 overexpression was sufficient to alleviate glomerular injury caused by diabetes, NCOA3 was overexpressed in kidneys with lentivirus. DKD was established as described above, and NCOA3 or control lentivirus was injected into kidneys directly 4 weeks before sacrifice (Figure S4A, Supporting Information). RT‐qPCR verified the transfection efficiency of NCOA3 lentivirus in mice, and mRNA analysis showed that NCOA3 levels were significantly increased in the glomeruli of NCOA3 lentivirus transfected mice (**Figure** **8**A). Kidney injury was detected in each group. The kidney size and weight were analyzed. NCOA3 overexpression relieved kidney hypertrophy (Figure 8B,C) and decreased UACR (Figure 8D) of DKD mice. The DKD mice treated with NCOA3 lentivirus exhibited improved glomerular filtration barrier, with specific manifestations including restored foot process width of podocytes, reduced GBM thickness, and increased number of foot processes compared with DKD mice injected with control lentivirus (Figure 8E–H). Glomerular mesangial expansion revealed by PAS staining was restored by NCOA3 overexpression (Figure 8I,J). Moreover, immunofluorescence staining of Synaptopodin and Nephrin, and immunohistochemical staining of WT1 revealed a reduction in the expression of Synaptopodin, Nephrin, and WT1 within the kidneys of DKD mice. Intriguingly, the overexpression of NCOA3 led to an augmentation in the expression levels of these markers (Figure S5A, S5B, Supporting Information). Correspondingly, the protein levels of Nephrin and Podocin were found to be reinstated, while Desmin levels were conversely suppressed in DKD mice treated with NCOA3 lentivirus, in comparison to DKD mice treated with the control lentivirus (Figure S5C,D, Supporting Information). The aforementioned findings suggested that overexpression of NCOA3 in DKD mice alleviated glomerular and podocyte injury caused by DKD.

We further examined the key regulator of autophagy, Fyn, and autophagy levels in the kidneys of DKD mice treated with NCOA3 overexpression lentivirus. The immunofluorescence of Fyn showed the elevated Fyn in DKD glomeruli was inhibited by NCOA3 overexpression (Figure 8K,L). The mRNA level and protein level of Fyn revealed by RT‐qPCR and Western blotting respectively displayed consistent results (Figure 8M–O). The Western blotting analysis demonstrated that NCOA3 overexpression in DKD mice led to an increase in phosphorylated AMPK, a decrease in phosphorylated mTOR (Figure 8P–R), and the restoration of autophagy‐associated proteins such as Atg5, Beclin1, p62, and LC3 (Figure 8S,T) compared with control DKD mice.

## 3 Discussion

The strong effect of NRs on regulating podocyte injury and DKD draws considerable attention. Their transcriptional activity can be powerfully boosted by binding to coactivators. However, the effects of coactivators of NRs in podocyte injury and DKD are neglected. In this study, we revealed the essential role of a member of the nuclear receptor coactivator family, NCOA3 in maintaining the physiological functions of podocytes. The expression of NCOA3 was decreased in the kidneys of DKD patients and mice. Podocyte‐specific *NCOA3* knockout enhanced albuminuria, glomerular and podocyte injury induced by diabetes. Overexpression of NCOA3 alleviated kidney injury in DKD mice, indicating that NCOA3 was a target that protected against podocyte injury. The potential mechanism was that NCOA3 improved podocyte autophagy by inhibiting Fyn transcription in the nuclear receptor, PPAR‐γ dependent way. The findings greatly enrich our understanding of the role of NCOA3 in DKD.

From the name of the NCOA family, we can easily remind of their close relationship with NRs. Indeed, NCOAs are one of the first coactivators found to interact with NRs.^[^ ^22^ ^]^ NCOAs are powerful coactivators of NRs due to three highly conserved LXXLL motifs which are essential for NRs interaction and activation.^[^ ^23^ ^]^ With the expansive research on coactivators of NRs, other coactivators like p300/ CBP, CARM1, and PRMT1 were identified. And NCOAs are also necessary for the interaction between these coactivators and NRs by providing a scaffold for further recruitment.^[^ ^16^ ^]^ Thus, we screened the expression of NCOAs in DKD kidneys and showed that only NCOA3 levels significantly differed from healthy kidneys. It indicated that NCOA3 rather than other NCOAs participated in the progression of DKD. We further confirmed the high basal level of NCOA3 in podocytes. Loss of NCOA3 induced spontaneous podocyte injury and glomerular sclerosis, indicating that NCOA3 deficiency was the driving force of podocyte damage.

NRs regulate numerous biological and pathological processes including autophagy which has been demonstrated to be indispensable in maintaining podocyte hemostasis.^[^ ^9^, ^24^, ^25^, ^26^ ^]^ In this study, we showed that overexpression of NCOA3 blunted podocyte injury by improving autophagy. Persistent activated Src kinase reduced autophagy in mTOR and AMPK dependent way.^[^ ^27^ ^]^ Through screening, a member of the Src kinase family, Fyn was shown to be downregulated by NCOA3 obviously. We demonstrated NCOA3 deficiency raised the transcription of Fyn, leading to decreased autophagy and podocyte injury. Fyn regulates multiple signals by phosphorylating signaling molecules and thus participates in physiological processes.^[^ ^28^, ^29^ ^]^ From the published papers, the effects of Fyn on kidney disease are consistent with our study. For example, activated Fyn augments oxidative stress, inflammation, endoplasmic reticulum stress, and autophagy dysfunction, resulting in the progression of acute kidney disease (AKI).^[^ ^30^ ^]^ Fyn which was upregulated in the DKD kidney promoted oxidative stress and impaired renal function.^[^ ^31^ ^]^ Fyn controls a group of genes that are associated with autophagy. Studies demonstrated that the absence of the Fyn gene leads to an increase in AMPK activity, which is dependent on the regulation of LKB1.^[^ ^32^, ^33^ ^]^ Recently research has revealed that the pro‐inflammatory factor TNF‐α activates Fyn kinase, which subsequently phosphorylates AMPKα, resulting in the inhibition of AMPK activity and suppression of autophagy.^[^ ^34^ ^]^ It was found that Fyn impeded macroautophagy by depleting Vps34 levels in a Stat3‐dependent manner, consequently diminishing the formation of the Vps34/p150/Beclin1/Atg14 complex.^[^ ^35^ ^]^ We assessed the downstream of Fyn that mediated autophagy, phosphorylation of AMPK and mTOR and found that NCOA3 overexpression promoted AMPK phosphorylation and inhibited mTOR phosphorylation. This is consistent with the previous reports that showed Fyn activated mTOR but inhibited the phosphorylation of AMPK.^[^ ^32^, ^36^ ^]^

We further investigated the mechanism of NCOA3 on Fyn regulation. And nuclear receptor, PPAR‐γ was identified to be the novel transcriptional factor of Fyn which interacted with NCOA3. The role of PPAR‐γ in podocytes has been well studied.^[^ ^15^, ^37^ ^]^ Podocyte‐specific *PPAR‐γ* knockout mice developed a more severe glomerulonephritis compared to wild‐type mice.^[^ ^38^ ^]^ PPAR‐γ agonist, pioglitazone significantly decreased puromycin induced podocyte apoptosis and necrosis.^[^ ^39^ ^]^ From another point of view, we reported a novel coactivator, NCOA3 of PPAR‐γ in this study. And we can get some supported information of this from adipocyte differentiation research. Double knockout of *NCOA1* and *NCOA3* impaired adipocyte adaptive thermogenesis due to failure in inducing the PPAR‐γ targeted genes.^[^ ^40^ ^]^ In addition, NCOA3 promotes human adipocyte differentiation by reducing phosphorylation of PPAR‐γ at S114 and modulating PPAR‐γ cellular heterogeneity.^[^ ^41^ ^]^ Our research showed PPAR‐γ to be the substrate of NCOA3. NRs beyond PPAR‐γ, like ERα, RAR, and non‐NRs transcription factors like NF‐κB, HIF1, AP‐1, and E2F1 have been reported to interact with NCOA3.^[^ ^42^, ^43^ ^]^ The diverse functions of NCOA3 on kidney diseases need further investigation.

To apply the findings in vivo, we established DKD models with podocyte *NCOA3* knockout mice and *NCOA3* overexpression lentivirus, respectively. The results showed loss of NCOA3 aggravated podocyte injury, albuminuria, and glomerular sclerosis by decreasing podocyte autophagy in Fyn dependent way. And NCOA3 supplement attenuated these changes in DKD mice. The role of NCOA3 on other diseases has been discussed. NCOA3 was initially found to be amplified in breast cancer and was reported to be abnormally overexpressed in various cancer types, including ovarian, esophageal, colorectal, and breast cancer.^[^ ^44^ ^]^ NCOA3 acts as an oncogene and its’ knockdown reduced the proliferation of nude mouse cancer cells and the growth of xenograft tumor cells, and increased cell apoptosis.^[^ ^45^, ^46^ ^]^ NCOA3 also takes part in metabolism diseases. Loss of NCOA3 protected against high‐fat diet (HFD)‐induced hepatic steatosis.^[^ ^47^ ^]^ The knockout mice are lean and resistant to obesity upon *NCOA3* knockout.^[^ ^48^ ^]^ Despite these harmful functions on cancer and liver diseases, loss of NCOA3 exhibited decreased growth and development, impaired neurologic, cardiac, and skeletal muscle performance.^[^ ^49^ ^]^ Considering the conflicting role of NCOA3 on different diseases, side effects have to be taken into account when investigating the prospective therapies targeted to NCOA3.

In summary, we focus on the key role of the coactivator of NRs in podocyte homeostasis and DKD and provide new insights into the effect of NCOA3 in kidney diseases. Our study raises the realization that coregulators of NRs play an important role in renal biology and pathology and represent a novel therapeutic target for DKD.

## 4 Experimental Section

### Human Kidney Biopsy Samples

Kidney biopsy samples were collected from the Department of Nephrology, Wuhan Union Hospital (Wuhan, China). Control kidney samples were obtained from healthy adults who underwent nephrectomy for kidney tumors without diabetes or any other kidney disease. All participants provided informed consent for the study. This study was approved by the Research Ethics Committee of Huazhong University of Science and Technology (UHCT230185)

### Animal Studies

All animal experiments were approved by the Animal Care and Use Committee of Tongji Medical College, Huazhong University of Science and Technology, Wuhan, China, and complied with the National Institutes of Health Guide for the Care and Use of Laboratory Animals (IACUC Number: 3143). To ensure unbiased results, groups were assigned randomly, and the researchers were blinded during the process of assigning groups for surgery and evaluating outcomes. The mice used in the study were all male, 7 weeks old, and housed in standard laboratory conditions, with a 12‐hour light‐dark cycle, a temperature of 21 ± 1 °C, and a humidity level of 50 ± 10%. They had ad libitum access to water and standard laboratory chow diet (Beijing Vital River Laboratory Animal Technology Co., Ltd., Beijing, China). The numbers of mice used in each experiment are indicated in the figure legends.

### Generation of Podocyte‐Specific *NCOA3* Knockout Mice

*NCOA3^fl/fl^* mice (C57BL/6J) were generated using standard homologous recombination and hybridized with mice expressing Cre recombinase (Cre) under the control of the NPHS promoter (B6. Cg‐Tg [NPHS2‐cre] 295Lbh/J; Stock No.008205, Jackson Laboratory, ME, USA) to generate podocyte‐specific *NCOA3* knockout mice (*Cre^+^/NCOA3^fl/fl^* mice). Mice lacking Cre expression were used as controls (*Cre^−^/NCOA3^fl/fl^* mice). Genotyping involving tail sample preparation and subsequent PCR was carried out. The primers utilized for flox genotyping were as follows: forward: 5′‐GCTAAGGCTGCACTGACAAT‐3′; reverse: 5′‐AGACACATAGCGAGCCAAGGAGAG‐3′. The primers utilized for Cre genotyping were as follows: forward: 5′‐GCGCTGCTGCTCCAG‐3′; reverse 5′‐ CGGTTATTCAACTTGCACCA‐3′

### High‐Fat‐Diet (HFD)/STZ‐Induced DKD in Mice

Seven‐week‐old *Cre^+^/NCOA3^fl/fl^* mice and littermate *Cre^−^/NCOA3^fl/fl^* mice were fed HFD (60 kcal%, D12492, Research Diets) or a control diet (10 kcal%, D12450J, Research Diets) for 4 weeks, and then unilateral nephrectomy (right kidney) was performed. One week after the operation, the mice underwent an 8‐hour fasting period, followed by the administration of low‐dose STZ (50 mg kg^−1^, 3 times a week, i.p.). The vehicle was injected as a control. The mice were maintained on HFD or control diets for the subsequent 12 weeks. Two weeks following STZ administration, the random blood glucose level was measured. And mice with glucose levels higher than 16.7 mmol L^−1^ were considered to have developed diabetes. To investigate the effects of AZD0530 (AZD0530 is diluted in 10% DMSO, 40% PEG300, 5% Tween‐80, and 45% Saline), another group of mice was administered with AZD0530 (20 mg kg^−1^ day^−1^) via irrigation through the stomach for 4 weeks before their sacrifice, the solvent was used in the control group. Urine samples were collected for biochemical analyses. The mice were euthanized. Blood and kidney samples were collected for subsequent analyses.

### DKD Model with NCOA3 Lentivirus (LV‐NCOA3)

Seven‐week‐old male C57BL/6J mice were purchased from Beijing Vital River Laboratory Animal Technology Co., Ltd. (Beijing, China). The DKD model was generated using the method described above. 5×10^6^ IU lentivirus in 100 µL cold sterile phosphate‐buffered saline (PBS) was injected slowly with 8 mm insulin needles into the superior and inferior poles of the kidney. After 4 weeks, urine and kidney samples were collected for analysis.

### Glomerulus Isolation

Mice were anesthetized and their hearts were perfused with 8×10^7^ Dynabeads (14 013, Invitrogen, Waltham, MA, USA) suspended in PBS. The kidney medulla and cortex were subsequently separated. The cortex was diced into 1mm^3^ pieces and digested with 2% collagenase (9001‐12‐1, Sigma Aldrich, MO, USA) for 15 min at 37 °C. The tissue was filtered through a 100 µm cell sieve, and the tissue that passed through the sieve was harvested and centrifuged (4 °C, 1500 rpm, 5 min). The pellet obtained was resuspended in PBS, and glomeruli that contained Dynabeads were separated using a magnetic particle concentrator and washed thrice with PBS. The enriched glomeruli precipitates were then collected for further analysis.

### Urine Albumin and Creatinine Measurement

Urine albumin and creatine levels were determined using commercial ELISA kits (ab108792, Abcam, Cambridge, UK) and DICT‐500 (Bioassay Systems, Hayward, CA, USA). The UACR was calculated as follows: UACR = (urine albumin) / (urine creatinine) × 1.73.

### Transmission Electron Microscopy (TEM)

Electron microscopy samples were handled and observed in the Department of Nephrology, Wuhan Union Hospital. Briefly, kidney tissues were cut into 1^3^ cm pieces and rapidly fixed using 2.5% glutaraldehyde at 4 °C. The samples were dehydrated using 50, 70, 80, 90, and 100% ethanol series, embedded in epoxy resin and hardener. The samples were sectioned, stained, and observed with an electron microscope. ImageJ software (National Institutes of Health, Bethesda, MD, USA) was used to calculate the number of foot processes per micrometer of the GBM, the foot process width, and the GBM thickness from the TEM images.

### Cell Culture, Transfection, Treatment, and siRNA Targeting

Conditionally immortalized human podocyte cell lines (HPCs) were originally provided by Dr. Saleem MA from University of Bristol at UK, and cultured as previously described.^[^ ^20^, ^50^ ^]^ HPCs were cultured in RPMI 1640 medium (Thermo, Grand Island, USA) containing 11.0 mmol L^−1^ glucose and 10% fetal bovine serum at 33 °C for proliferation and then shifted to 37 °C for 10 days to induce differentiation. Overexpression of NCOA3 with NCOA3 lentivirus, inhibition of NCOA3 by si‐NCOA3 (target sequence 5′‐3′: GGAGAATAATGCACTTCTT), inhibition of PPAR‐γ by si‐PPAR‐γ (target sequence 5′‐3′: GCAATTGAATGTCGTGTCT), and inhibition of Fyn via si‐Fyn (target sequence 5′‐3′: GGTGGATACTACATTACCA) was conducted in differentiated podocytes. The stimuli used in this study were: (1) high glucose (HG, 30 mmol L^−1^ glucose; 49 163; Sigma‐Aldrich, St Louis, MO, USA) for 6, 12, and 24 h in culture medium, medium contained 5.6 mmol L^−1^ glucose as control group, mannitol was added in the medium as the osmotic pressure control for HG; (2) advanced glycation end products (AGE, 50, 100, 150 µg mL^−1^, ab51995, Abcam, Cambridge, UK).

### RNA Extraction and Reverse Transcription‐Quantitative PCR (RT‐qPCR)

RT‐qPCR was conducted as previously reported.^[^ ^51^ ^]^ Briefly, TRIzol reagent was used to extract RNA from the kidney samples, and cDNA was synthesized using the HiScript II Reverse Transcription System (Vazyme, Nanjing, China). RT‐qPCR analyses were conducted using the ChamQ SYBR Color qPCR Master Mix kit (Vazyme) on a Bio‐Rad Cycler system (Bio‐Rad, Hercules, CA, USA). The primer sequences are given in Table S2 (Supporting Information).

### Western Blotting

Western blotting was conducted as previously reported.^[^ ^51^ ^]^ To briefly summarize, protein lysates were prepared from cultured cells and kidney tissues using RIPA lysis buffer supplemented with protease inhibitor cocktails, phenylmethylsulfonyl fluoride, and phosphatase inhibitor. After sodium dodecyl sulfate‐polyacrylamide gel electrophoresis, samples were transferred to polyvinylidene difluoride membranes. The membranes were then blocked with 5% milk powder, and then incubated with primary antibodies (Table S3, Supporting Information). For loading controls, the membranes were re‐tested with a primary antibody against the housekeeper protein GAPDH, β‐actin, and α‐tubulin.

### Co‐Immunoprecipitation (Co‐IP)

The interaction between NCOA3 and PPAR‐γ was determined using Co‐IP. HPCs were subjected to lysate on ice for 30 min, followed by centrifugation at 12 000 rpm for 15 min at 4 °C. The supernatant was collected and incubated with anti‐NCOA3 antibody overnight at 4 °C and protein A/G agarose (Beyotime Biotechnology, Shanghai, China) for 3 h at 4 °C. The mixtures were then washed with cold PBS, and precipitated proteins were collected for Western blotting with anti‐NCOA3 and anti‐PPAR‐γ antibodies (Table S3, Supporting Information).

### Chromatin Immunoprecipitation (ChIP)

ChIP was performed with the ChIP assay kit (P2078, Beyotime Biotechnology, China). In short, 1% methanal was added to the culture medium to fix cells, and added 1× Glycine Solution and washed with 1×PBS and 1% PMSF according to the instructions. Collected cells and resuspended with SDS Lysis buffer. DNA was crushed by ultrasonic equipment. Diluted DNA fragments with ChIP dilution buffer and 20 µL of the sample was collected as input. Other samples were incubated with Protein A+G Agarose/Salmon Sperm DNA and 2 µg primary antibody overnight on a rotating table at 4°C. Then, the samples were washed and purified for PCR amplification and DNA gel electrophoresis. The primer sequences are given in Table S2 (Supporting Information).

### Periodic‐Acid‐Schiff (PAS) Staining

PAS staining was performed by using staining kits (Solarbio, Beijing, China) according to the manufacturer's instructions. The grading of renal glomerular injury was classified based on the extent of mesangial expansion and glomerular enlargement in the glomeruli. Semiquantitation of staining was performed in a blinded fashion. The grading system is as follows: Grade 0: This grade indicates the absence of mesangial expansion and glomerular enlargement in all glomeruli. Grade 1: In this grade, there is evidence of mesangial expansion and/or glomerular enlargement affecting 0–25% of the normal glomeruli. Grade 2: Mesangial expansion and/or glomerular enlargement are observed in 25–50% of the normal glomeruli in this grade. Grade 3: Mesangial expansion and/or glomerular enlargement affect 50–75% of the normal glomeruli in this grade. Grade 4: The highest grade represents mesangial expansion and/or glomerular enlargement affecting 75–100% of the normal glomeruli. To calculate the renal injury index for each kidney tissue sample, the following formula is utilized: (N1×1+ N2×2+ N3×3+ N4×4)/n, where N1, N2, N3, and N4 represent the number of glomeruli exhibiting Grades 1, 2, 3, and 4 injury, respectively. The variable n corresponds to the total number of observed glomeruli under a 40x objective lens.

### Immunohistochemistry Staining

Kidney samples were fixed with 4% paraformaldehyde and embedded in paraffin. Sections (3 µm) were dewaxed and rehydrated for immunohistochemistry staining. Endogenous peroxidases were blocked with 3% H_2_O_2_ for 15 min, and non‐specific antibodies were incubated with 5% bovine serum at room temperature for 1 h after antigen retrieval. Next, the sections were incubated with primary antibodies overnight at 4 °C, and then with a secondary antibody for 1 h at room temperature, and HRP‐labeled streptavidin was incubated for 45 min. Finally, peroxidase activity was observed using 3′,3′‐diaminobenzidine, and the sections were stained with hematoxylin before the images were obtained. The staining was quantified using ImageJ software by measuring the positive area for each glomerulus.

### Immunofluorescence Staining

Kidney sections were prepared and immunofluorescence staining was performed as described previously.^[^ ^51^ ^]^ Images were obtained using an LSM780 confocal laser scanning microscope system (ZEISS, Oberkochen, Germany).

### Flow Cytometry

Annexin V/FITC staining was performed according to the manufacturers’ instructions by using Annexin V/FITC kit (Beyotime Biotechnology, Shanghai, China). HPCs treated with HG and/or LV‐NCOA3 were harvested and resuspended in PBS. After centrifugation, FITC‐conjugated annexin V binding buffer and annexin V‐FITC in the kit were added. The mixtures were then incubated at room temperature in the dark and centrifuged at 1000 *g* for 5 min, and FITC‐conjugated annexin V buffer and propidium iodide were added. The samples were analyzed using a spectral cell analyzer (ID7000, Sony Biotechnology, San Jose, CA, USA). The percentage of apoptotic cells in each sample was calculated with FlowJo software (FlowJo, LLC, Ashland, OR, USA).

### Statistics

Experiments were repeated at least thrice, and representative outcomes were shown. Data are presented as mean ± standard error of the mean (SEM). Data were analyzed and plotted using GraphPad Prism (GraphPad Software, San Diego, CA, USA). Student's *t*‐test was used to compare two experimental groups, and one‐way ANOVA followed by Tukey's post‐hoc test for multiple comparisons was used to compare three or more groups. All tests were two‐tailed.

### Ethics Statement

All the animal experiments were performed with the approval by Ethics Committee of Huazhong University of Science and Technology and conducted following National Institutes of Health (NIH) guidelines for the use and care of laboratory animals. All participants provided informed consent for the study and his study was approved by the Research Ethics Committee of Huazhong University of Science and Technology.

## Conflict of Interest

The authors declare no conflict of interest.

## Author Contributions

Y.X. and Q.Y. contributed equally to this work. YR.X., Q.Y., XF.M., and C.Z. designed the study. YR.X. and Q.Y. collected and analyzed the data. YR.X., Y.Q., JY.Z., and YL.C. performed animal models. YR.X., YJ.X., and XY.C. performed in vitro experiments. YR.X. and Q.Y. wrote the paper. XF.M., K.H., and F.Y. revised the manuscript. C.Z. approved the final version of the manuscript. All authors read and approved the final paper.

## Supporting information

Supporting Information

## Data Availability

Data sharing is not applicable to this article as no new data were created or analyzed in this study.
