# Supplementary material for: Deficiency of Nuclear Receptor Coactivator 3 Aggravates Diabetic Kidney Disease by Impairing Podocyte Autophagy
Source: Adv Sci (Weinh). 2024 Mar 14;11(19):2308378. doi: 10.1002/advs.202308378 (PMC11109634; doi:10.1002/advs.202308378)
Supplement: Supplementary file 1 — Supporting Information [file ADVS-11-2308378-s001.pdf]

ADVANCED SCIENCE
Open Access

Supporting Information

for *Adv. Sci.*, DOI 10.1002/advs.202308378

Deficiency of Nuclear Receptor Coactivator 3 Aggravates Diabetic Kidney Disease by Impairing Podocyte Autophagy

*Yaru Xie, Qian Yuan, Xinyi Cao, Yang Qiu, Jieyu Zeng, Yiling Cao, Yajuan Xie, Xianfang Meng, Kun Huang, Fan Yi and Chun Zhang**

Supporting Information

Deficiency of Nuclear Receptor Coactivator 3 aggravates Diabetic Kidney Disease by impairing podocyte autophagy

*Yaru Xie, Qian Yuan, Xinyi Cao, Yang Qiu, Jieyu Zeng, Yiling Cao, Yajuan Xie, Xianfang Meng, Kun Huang, Fan Yi, Chun Zhang**

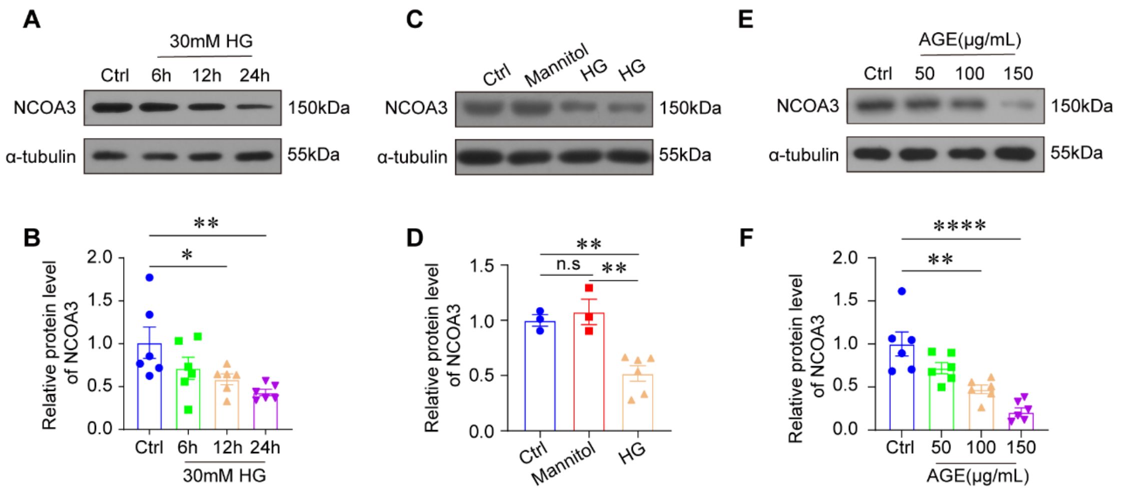

Figure S1 | NCOA3 significantly reduced *in vitro* with HG and AGE stimulation. **(A, B)** Representative Western blotting **(A)** and quantification **(B)** showing the relative protein level of NCOA3 in HPCs with HG (30mM) stimulation in a time-dependent manner (6, 12, 24 hours). n = 6 per group. **(C, D)** Representative Western blotting **(C)** and quantification **(D)** of NCOA3 expression in HPCs under HG (30 mM) conditions for 24 hours, mannitol was added as the osmotic pressure control for HG (n = 3 or 6 per group). **(E, F)** Representative Western blotting **(E)** and quantification **(F)** showing the protein level of NCOA3 in HPCs with AGE stimulation in a dose-dependent manner (50, 100, 150 μg/mL). n = 6 per group. * $P < 0.05$, ** $P < 0.01$, **** $P < 0.0001$. n.s, no significance. Data are expressed as means ± SEM. One-way

ANOVA was applied to the comparison between groups.

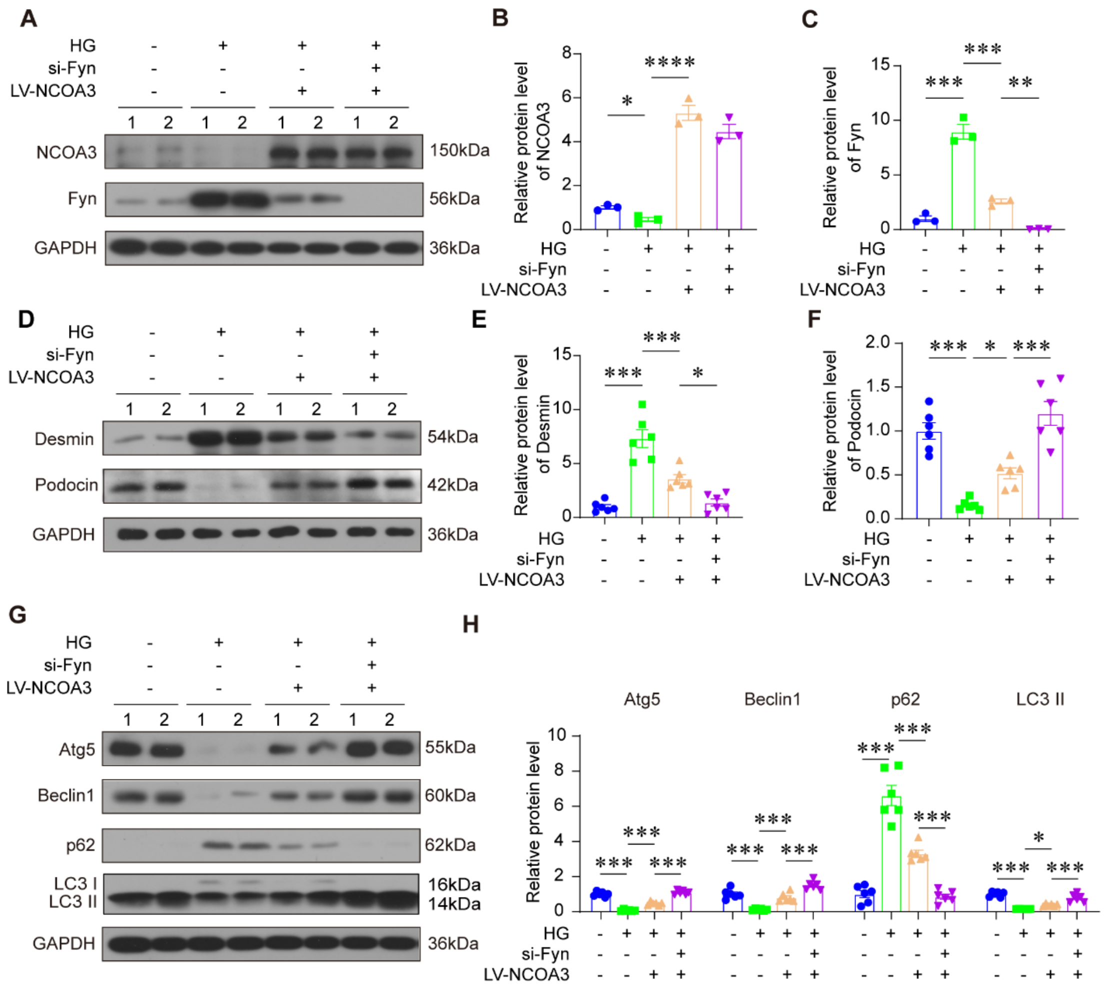

Figure S2 | NCOA3 promotes autophagy by suppressing Fyn expression. (A-C) Representative Western blotting **(A)** and quantification of NCOA3 **(B)** and Fyn **(C)** in HPCs with LV-NCOA3 and si-Fyn transfection under HG stimulation (n = 3 per group). **(D-F)** Representative Western blotting **(D)** and quantification of Desmin **(E)** and Podocin **(F)** from different groups (n = 6 per group). **(G, H)** Representative Western blotting **(G)** and quantification of Atg5, Beclin1, p62, and LC3 **(H)** from different groups (n = 6 per group). * $P < 0.05$, ** $P < 0.01$, *** $P < 0.001$. Data are expressed as mean ± SEM. One-way ANOVA was applied for comparisons.

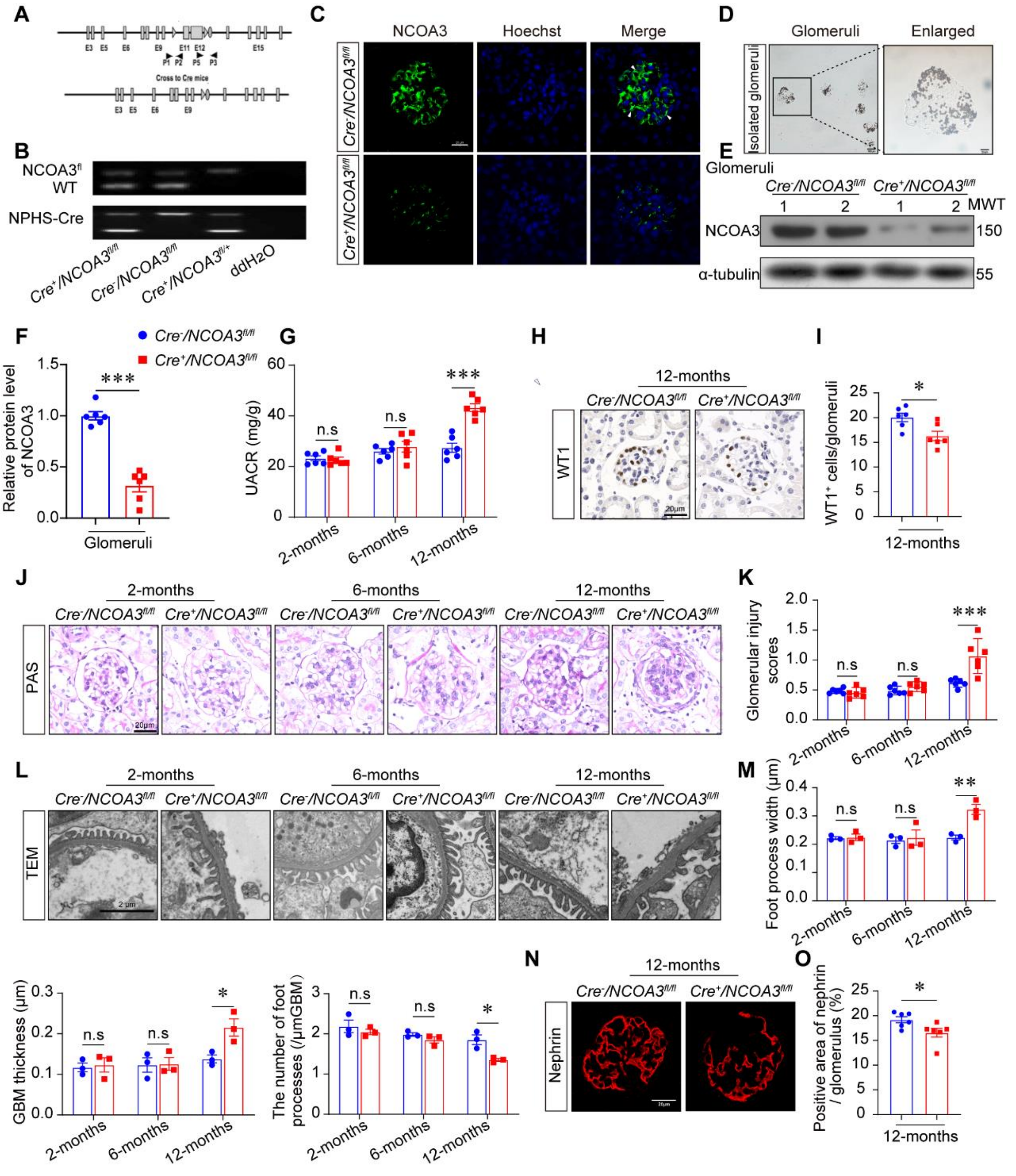

Figure S3 | Establishment of podocyte-specific *NCOA3* knockout (*Cre*$^{+}$/*NCOA3*$^{fl/fl}$) mice. (A) Construction of podocyte-specific NCOA3 knockout mice using the Cre-LoxP system. **(B)** Genotyping was confirmed using PCR on tail samples. **(C)** Representative immunofluorescence staining images of NCOA3 (green). Scale bar, 20 μm. **(D)** Isolated glomeruli captured by microscopy. Scale bar, 100 μm (left), 20 μm (right). **(E, F)** Representative Western blotting **(E)** and quantification **(F)** of NCOA3 expression in glomeruli from *Cre*$^{+}$/*NCOA3*$^{fl/fl}$ and *Cre*$^{-}$/*NCOA3*$^{fl/fl}$ mice (n = 6 per

group). MWT was the abbreviation of molecular weight. **(G)** UACR in mice of different ages (n = 6). **(H, I)** Representative photomicrographs **(H)** and quantification **(I)** of WT1 in the kidney from different groups at the age of 12 months showing the number of podocytes in each glomerulus (15 glomeruli per mouse, n = 6 mice per group). Scale bar, 20 μm. **(J, K)** Representative photomicrographs **(J)** and quantification **(K)** of periodic acid-Schiff (PAS) staining showing typical glomerular structures of mice in different groups at different ages (15 glomeruli per mouse, n = 6 mice per group). Scale bar, 20 μm. **(L, M)** Morphological changes in podocyte foot processes in mice in different groups and at different ages were analyzed using transmission electron microscopy (TEM) showing the foot process width, GBM thickness, and the number of foot processes (15 glomeruli per mouse, n = 3 mice per group). Scale bar, 2 μm. **(N, O)** Representative immunofluorescence staining images **(N)** and quantification **(O)** showing expression of Nephrin in the kidney in different groups at the age of 12 months (15 glomeruli per mouse, n = 6 mice per group). Scale bar, 20 μm. * $P < 0.05$, ** $P < 0.01$, *** $P < 0.001$, n.s, no significance. Data are expressed as mean ± SEM. One-way ANOVA was applied for comparisons.

A

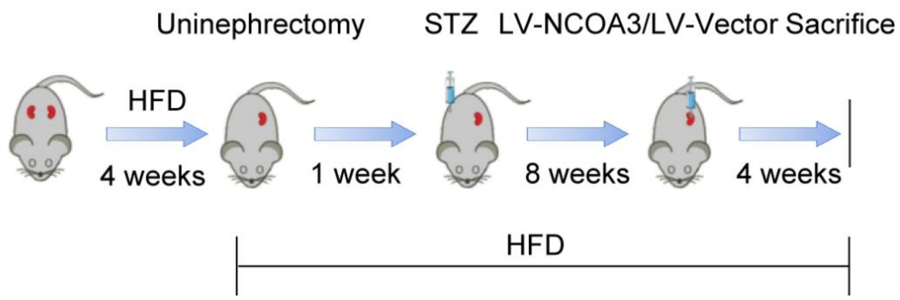

Figure S4 | Schematic diagram of DKD model with lentivirus.

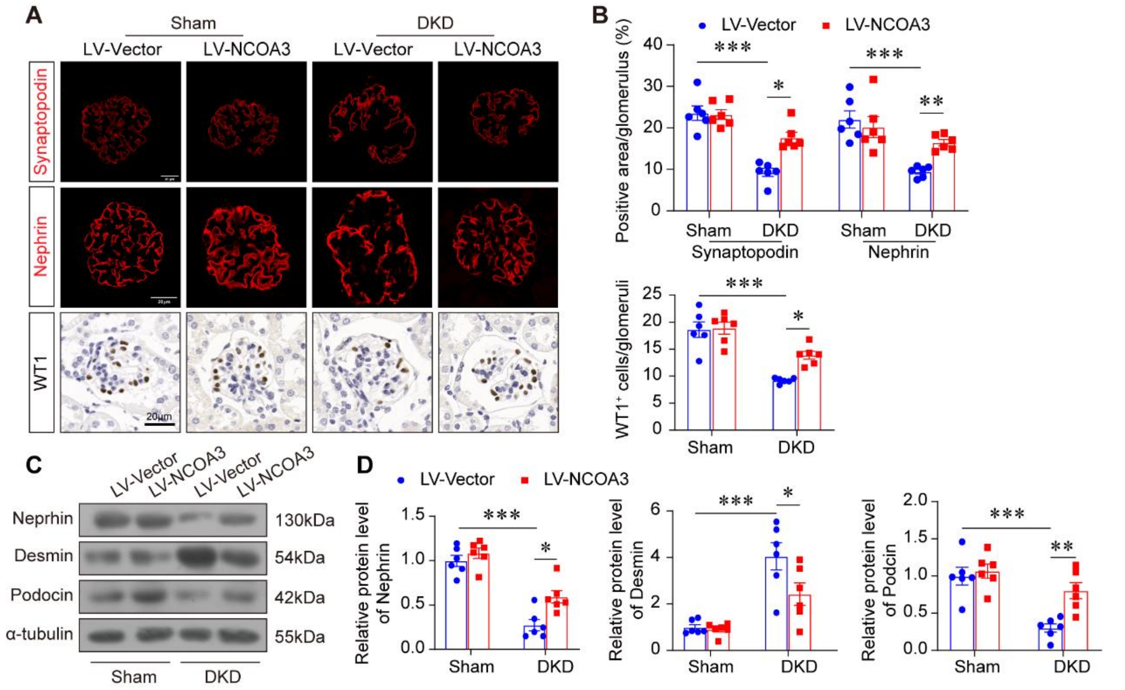

Figure S5 | NCOA3 overexpression attenuated glomerular and podocyte injury in DKD mice. (A, B) Representative immunofluorescent staining micrographs **(A, top and medium)**, immunohistochemistry staining **(A, bottom)**, and quantification **(B)** showing the expression of Synaptopodin, Nephrin, and WT1 in the kidney from different groups (15 glomeruli per mouse, n = 6 mice per group). Scale bar, 20 μm. **(C, D)** Representative Western blotting **(C)** and quantification **(D)** of Nephrin, Desmin, and Podocin from different groups (n = 6 per group). * *P* < 0.05, ** *P* < 0.01, *** *P* <0.001. Data are expressed as mean ± SEM. One-way ANOVA was applied for comparisons.

Table S1 Body weight and blood glucose in STZ/HFD-induced $Cre^{+}/NCOA3^{fl/fl}$ mice and $Cre^{-}/NCOA3^{fl/fl}$ mice.

Variables	Ctrl		DKD	
	$Cre^{-}/NCOA3^{fl/fl}$	$Cre^{+}/NCOA3^{fl/fl}$	$Cre^{-}/NCOA3^{fl/fl}$	$Cre^{+}/NCOA3^{fl/fl}$
BW (g)	24.35±2.886	22.683±2.596	24.10±0.676	22.533±2.815
KW (g)	0.301±0.045	0.289±0.038	0.360±0.011	0.476±0.031
Blood glucose (mmol/l)	6.05±0.377	6.25±0.377	29.367±1.521	29.65±1.799

Table S2 Primers for RT-qPCR and PCR

Species	gene	Forward (5' to 3')	Reverse (5' to 3')
Mouse	*NCOA1*	TATCTCTCCAGCCCATGGTGT	CAAAGTTCCCTTGGTTGTTGC
Mouse	*NCOA2*	AGCAGTTCCCATTTCCTCCG	GCCATCCGGGGAGACATTAG
Mouse	*NCOA3*	CTGTGCCTACCTTGCCACTTCG	GCTGCTGTTGCTGCTGTTGTTG
Mouse	*Fyn*	AAGGATAAAGAAGCAGCGAAAC	TGCGTGGAAGTTGTTGTAGTTC
Mouse	*β-actin*	GGACTCCTATGTGGGTGACGAG	TCACGGTTGGCCTTAGGGTT
Human	*Src*	TGTTCGGAGGCTTCAACTCC	TGTTCGGAGGCTTCAACTCC
Human	*Lyn*	AAGACTCAACCAGTTCCAGAATC	AAGACTCAACCAGTTCCAGAATC
Human	*Fyn*	GTACGAGAGGAGGAACAGGAGTG	GTCATCTTCTGTCCGTGCTTCATAG
Human	*Lck*	GCTATGAGCCCTCTCACGAC	GTAGAAGCCACCGTTGTCCA
Human	*Blk*	AGTTGTCACTTATGGGCGGG	AGTTGTCACTTATGGGCGGG
Human	*Hck*	AGGGCTACATCCCAAGCAAC	AGGGCTACATCCCAAGCAAC
Human	*Yes*	TAGCGCCTGCAGATTCCATT	TAGCGCCTGCAGATTCCATT
Human	*Fgr*	CTTCACCAAGGGCGAGAAGT	CAGGGAGTAGGCACCTTTGG
Human	*PPARG*	GCCGAGAAGGAGAAGCTGTTG	GCCGAGAAGGAGAAGCTGTTG
Human	ChIP primer1	TAAAGACCGATGCACTCCCA	CGGCGACTAAGGAACCTGAA
Human	ChIP primer2	AAATCACCCTTCTTGCCCCT	CCCAGGGAAGAGAGACAAGA

Table S3 List of antibodies used in the current study

Primary antibodies	Host	Dilution and supplier	Application
NCOA1	Mouse	1:1,000; Santa Cruz, Dallas, US	WB
NCOA2	Rabbit	1:1,000; Abcam, Cambridge, GB	WB
NCOA3	Rabbit	1:1,000; Abcam, Cambridge, GB	WB, IF, IHC
Synaptopodin	Guinea pig	1:400; Synaptic Systems, Göttingen, DE	IF
Nephrin	Goat	1:2,000; R&D Systems, Minnesota, US	IF, WB
Podocin	Rabbit	1:2,000; Sigma, St. Louis, US	IF, WB
Desmin	Rabbit	1:1,000, Bioworld Technology, Minnesota, US	IF, WB
WT1	Rabbit	1:400; Abcam, Cambridge, GB	IHC
Fyn	Mouse	1:5,000; ProteinTech Group, Chicago, US	IF, WB
AMPK	Rabbit	1:1,000; Cell signaling, Danvers, US	WB
T172-AMPK	Rabbit	1:1,000; Cell signaling, Danvers, US	WB
mTOR	Rabbit	1:1,000; Cell signaling, Danvers, US	WB
S2448-mTOR	Rabbit	1:1,000; Cell signaling, Danvers, US	WB
Atg5	Rabbit	1:1,000; Cell signaling, Danvers, US	WB
Beclin1	Rabbit	1:1,000; ProteinTech Group, Chicago, IL	WB
SQSTM1/p62	Rabbit	1:1000; ABclonal, Wuhan, CN	WB
LC3	Rabbit	1:1,000; ProteinTech Group, Chicago, IL	WB
PPAR-γ	Rabbit	1:1,000; ProteinTech Group, Chicago, IL	WB
PPAR-γ	Mouse	1:2,000; ProteinTech Group, Chicago, IL	WB
α-tubulin	Rabbit	1:2,000; ProteinTech Group, Chicago, IL	WB
β-actin	Rabbit	1:2,000; ProteinTech Group, Chicago, IL	WB
GAPHD	Rabbit	1:2,000; ProteinTech Group, Chicago, IL	WB
